# Safety and effectiveness of opioid-free anaesthesia in thoracoscopic surgery: a preliminary retrospective cohort study

**DOI:** 10.1186/s12871-024-02441-9

**Published:** 2024-02-09

**Authors:** Shanshan Zhang, Jianmin Zhang, Ran Zhang

**Affiliations:** 1 Department of Anesthesiology, Beijing Children’s Hospital, National Center for Children’s Health, Capital Medical University, Beijing, China; 2 Department of Anesthesiology and Pain Medicine, Peking University People’s Hospital, No.11 Xizhimen South Street, Xicheng District 100044, Beijing, China

**Keywords:** Intraoperative haemodynamics, Opioid-free anaesthesia, Thoracoscopic surgery, Postoperative analgesia

## Abstract

**Background:**

This study aimed to observe the effect of opioid-free anaesthesia (OFA) on intraoperative haemodynamic,postoperative analgesia and postoperative nausea and vomiting (PONV) in thoracoscopic surgery in order to provide more evidence for evaluating the safety and effectiveness of OFA technology.

**Methods:**

This was a single-centre retrospective observational study.Adult patients who underwent thoracoscopic surgery with the preoperative thoracic paravertebral block between January 2017 and June 2020 were included.A cohort of 101 thoracoscopic surgery patients who received the OFA technique were matched with 101 thoracoscopic surgery patients who received standard opioid-containing anaesthesia(SOA). Heart rate (HR) and mean arterial blood pressure (MAP) were measured before anaesthesia induction, immediately after endotracheal intubation, at the beginning of surgery, and 10, 20, and 30 min after surgery began.The total amount of intraoperative infusion, frequency of vasoactive drugs use, morphine ingested via the patient-controlled intravenous analgesia (PCIA) 24 h post-surgery,visual analogue scale (VAS) scores at rest and activity on the first day post-surgery, and frequency of nausea and vomiting within 24 h post-surgery were analysed.

**Results:**

There was no significant difference in intraoperative HR between the two groups (F = 0.889, *P* = 0.347); however, there was significant difference in intraoperative MAP (F = 16.709, *P* < 0.001), which was lower in SOA patients than in OFA patients. The frequency of vasoactive drug use and amount of infusion was less in OFA patients (*P* = 0.001). The consumption of morphine used by the PCIA 24 h post-surgery was significantly lower in OFA patients (OFA, 1.8 [0, 4.8] mg vs. SOA, 3.6 [0.6, 23] mg, *P* < 0.001). There was no significant difference in VAS scores at rest (*P* = 0.745) or during activity (*P* = 0.792) on the first day post-surgery. There was also no statistically significant difference in nausea and vomiting within 24 h post-surgery (*P* = 0.651).

**Conclusions:**

This case-control study demonstrated that compared with SOA, OFA can effectively maintain the stability of intraoperative MAP, reduce the incidence of hypotension. Although OFA reduced morphine consumption via the PCIA pump 24 h post-surgery, postoperative pain scores and nausea and vomiting within 24 h post-surgery were similar between the groups.But this study was only a preliminary study and needed to confirm in a larger, more robust trial.

## Introduction

Owing to its advantages of minimal trauma, quick recovery, and short hospital stay [1], thoracoscopic surgery has undergone rapid development in clinical practice; consequently, the demand for analgesia has decreased. However, postoperative pain following thoracoscopic surgery is still severe. In recent years, the concept of enhanced recovery after surgery (ERAS) has been promoted and applied in many surgical fields, including general surgery and orthopaedics [2]. During the implementation of ERAS, perioperative pain control is the most important goal, primarily through the use of opioids [3]. However, opioids are not without drawbacks. While they control pain, they also produce many adverse reactions, including nausea, vomiting [4], gastrointestinal peristalsis inhibition [5], urinary retention, respiratory inhibition, pain sensitization [6, 7], and tolerance. Reducing the use of perioperative opioids reduces the incidence of perioperative adverse reactions and facilitates rapid postoperative recovery. Therefore, reduction of opioid dosage and the development of de-opiating opioids have become research hotspots in recent decades [8–10].

Opioid-free anaesthesia (OFA) is a multi-mode anaesthesia that combines sedatives, N-methyl-D-aspartate antagonists [11], local anesthetics, anti-inflammatory drugs, α-2 agonists, and regional block techniques [7, 12]. Previous reports have demonstrated that OFA is feasible and effective compared with traditional opioid-based anaesthesia [13]. However, there are few studies on the application of OFA in thoracoscopic surgery. Therefore, patients undergoing thoracoscopic surgery in the Department of Thoracic Surgery at our hospital were selected as the research participants in this study. We reviewed the effects of OFA and traditional opioid-containing anaesthesia on intraoperative haemodynamic changes,postoperative analgesia and postoperative nausea and vomiting, and aimed to provide more evidence evaluating the safety and efficacy of OFA techniques.

## Methods

### Patient selection and study design

This was a single-centre retrospective observational study.This study was approved by our institutional review board and informed consent was obtained (2020PHB308-01).Patients who underwent thoracoscopic surgery between January 2017 and June 2020 were retrospectively analyzed. The exclusion criteria was as follows: (1) aged < 18 years; (2) did not receive preoperative nerve block or received other nerve blocks (excluding thoracic paravertebral nerve block; TPVB); (3) did not receive postoperative patient-controlled intravenous analgesia (PCIA); (4) transferred to the intensive care unit (ICU) after surgery; (5) those who did not receive follow-up after surgery; and (6) incomplete data.

The primary objective was to compare the stability of intraoperative haemodynamic changes between two groups of patients (OFA and standard opioid-containing anaesthesia (SOA) group)by collecting heart rate (HR) and mean arterial blood pressure (MAP) measurements at six time points (before anaesthesia induction, immediately after endotracheal intubation, beginning of surgery, and 10, 20, and 30 min after the beginning of surgery).

The secondary objectives included the total amount of intraoperative infusion, frequency of vasoactive drugs use, morphine ingested via the PCIA 24 h post-surgery, and visual analogue scale (VAS) scores at rest and activity on the first day post-surgery, and the frequency of nausea and vomiting 24 h post-surgery.

### Anaesthesia method and management

All patients received ultrasound-guided TPVB at the T3 and T6 segments of the surgical side before surgery, and 15 mL of 0.4% ropivacaine was injected into the thoracic paravertebral space at the corresponding segments. With the diffusion of the drug solution, the pleura was evidently depressed, which confirmed that the local anesthetic was well diffused. The blocking effect was determined by acupuncture 15 min after the start of the procedure. Successful thoracic paravertebral block using the two-point method was confirmed when the head side reached T3 or higher, and the tail side reached T8 or lower. If the above-mentioned levels were not reached, remediation and re-evaluation were performed. If this failed, the block was considered a failure, and the patient was excluded from the analysis.

Patients in the OFA group were given a 1 µg/kg load dose of dexmedetomidine hydrochloride by intravenous pump within 15 min of entering the operating room; subsequently, a rate of 0.4 µg/kg/h was maintained until 30 min before the end of surgery. Before anaesthesia induction, 0.5% tetracaine hydrochloride was used for laryngeal surface anaesthesia, and 0.2–0.4 mg/kg etomidate, 1.5–2 mg/kg lidocaine,and 1 mg/kg esmolol were injected intravenously to obtund the pressor response to intubation in the absence of opioids. During the operation, propofol was continuously administered intravenously to maintain the bispectral index (BIS) value between 40–60.Lidocaine (1.0–1.5 mg/kg) was injected intravenously to maintain the analgesic effect.

Patients in the SOA group did not receive any intravenous lidocaine. Anaesthesia was induced using propofol 1.5-2 mg/kg or etomidate 0.2–0.3 mg/kg, cisatracurium 0.3–0.4 mg/kg or rocuronium 0.5–0.6 mg/kg, and sufentanil 0.2–0.4 µg/kg and/or remifentanil 0.5-2 µg/kg. Anaesthesia was maintained using propofol and remifentanil. The propofol dosage was adjusted during the procedure to maintain the BIS between 40 and 60, and muscle relaxants were applied according to the muscle relaxation interval. Additional sufentanil was administered in appropriate doses according to the changes in the HR and blood pressure during surgery. After induction of anaesthesia, double-lumen endotracheal intubation was performed in both groups to achieve one-lung ventilation.

Based on the premise of maintaining satisfactory anaesthesia depth, if hypotension occurred (MAP decrease ≥ 20% of the basic value or MAP < 60 mmHg), 6 mg intravenous ephedrine or 50 µg deoxyepinephrine was injected intravenously. If bradycardia (HR < 50 times/min) occurred for 1 min, 0.5 mg atropine was injected intravenously.

PCIA was used for postoperative analgesia, using 50 mg oxycodone plus normal saline to 100 mL without a background dose. PCIA was delivered in 2 mL doses with an interval of 5 min. At the end of the operation, all patients in both groups were transferred to the postanesthesia care unit (PACU) with tracheal intubation and the PCIA infusion started.

Postoperative extubation criteria was as follows: the patient regained spontaneous breathing, tidal volume ≥ 5 mL/kg, respiratory rate < 20 times/min, fingertip oxygen saturation ≥ 95%, could move according to instructions, body temperature ≥ 36 ℃, and was routinely treated with muscle relaxant antagonists (atropine + neostigmine) before extubation. After extubation the 24 h observation period started.

Regarding postoperative treatment measures, after extubation, patient’s pain situation was evaluated. If the numerical rating scale score was greater than three points, oxycodone or sufentanil treatment was provided, according to the standard process.If the Aldrete score was > 9 points, the patient was returned to the general ward.

### Data collection

Data was collected by consulting the electronic medical record system. The data collected included general patient information; HR and MAP before anaesthesia (T0), immediately after endotracheal intubation (T1), at the beginning of surgery (T2), and 10 min (T3), 20 min (T4), and 30 min (T5) after surgery began.

Additionally,intraoperative infusion volume and frequency of vasoactive drugs; dosage of opioids in the PCIA pump 24 h post-surgery (expressed by equivalent morphine dose: 1 mg oxycodone = 1.5 mg morphine, 1 ug sufentanil = 1 mg morphine); and VAS score at rest and during activity on the first day post-surgery,and nausea and vomiting 24 h post-surgery were collected. Nausea was subjectively evaluated by the patient themselves; vomiting was divided into dry vomiting or vomiting, and the frequency was recorded in time periods. Within a short period, multiple times of dry vomiting or vomiting were only recorded once. If severe nausea and vomiting occurred, intravenous antiemetic medication was administered. All participants were followed up by professionally trained nurse anesthetists, and the data were recorded in an electronic database.

### Statistical analysis

SPSS 25.0 software was used for data analysis. The patients were included in the logistic model tendency score according to relevant covariables including age, sex, American Society of Anesthesiologists (ASA) grade, body mass index (BMI), presence or absence of hypertension, surgical type, and intraoperative blood loss. Nearest-neighbor matching (NNM) was adopted, the caliper value was set at 0.1, and matching was performed in a 1:1 ratio. Continuous data were tested for normality with the Shapiro–Wilk test. Continuous variables with normal distribution are presented as mean ± standard deviation (SD) and the independent sample t-test was used for inter-group comparisons. Non-normal variables are reported as median (interquartile range) and the Kruskal–Wallis H test was used for comparison between groups. The counting data are expressed as percentage (%), and the chi-square test was used for comparison between groups. The Kruskal–Wallis H test was used for grade data. A repeated-measurement analysis of variance was used for data of repeated measurements of the same index between groups. *P* < 0.05 was considered statistically significant difference.

## Results

**Fig. 1:**
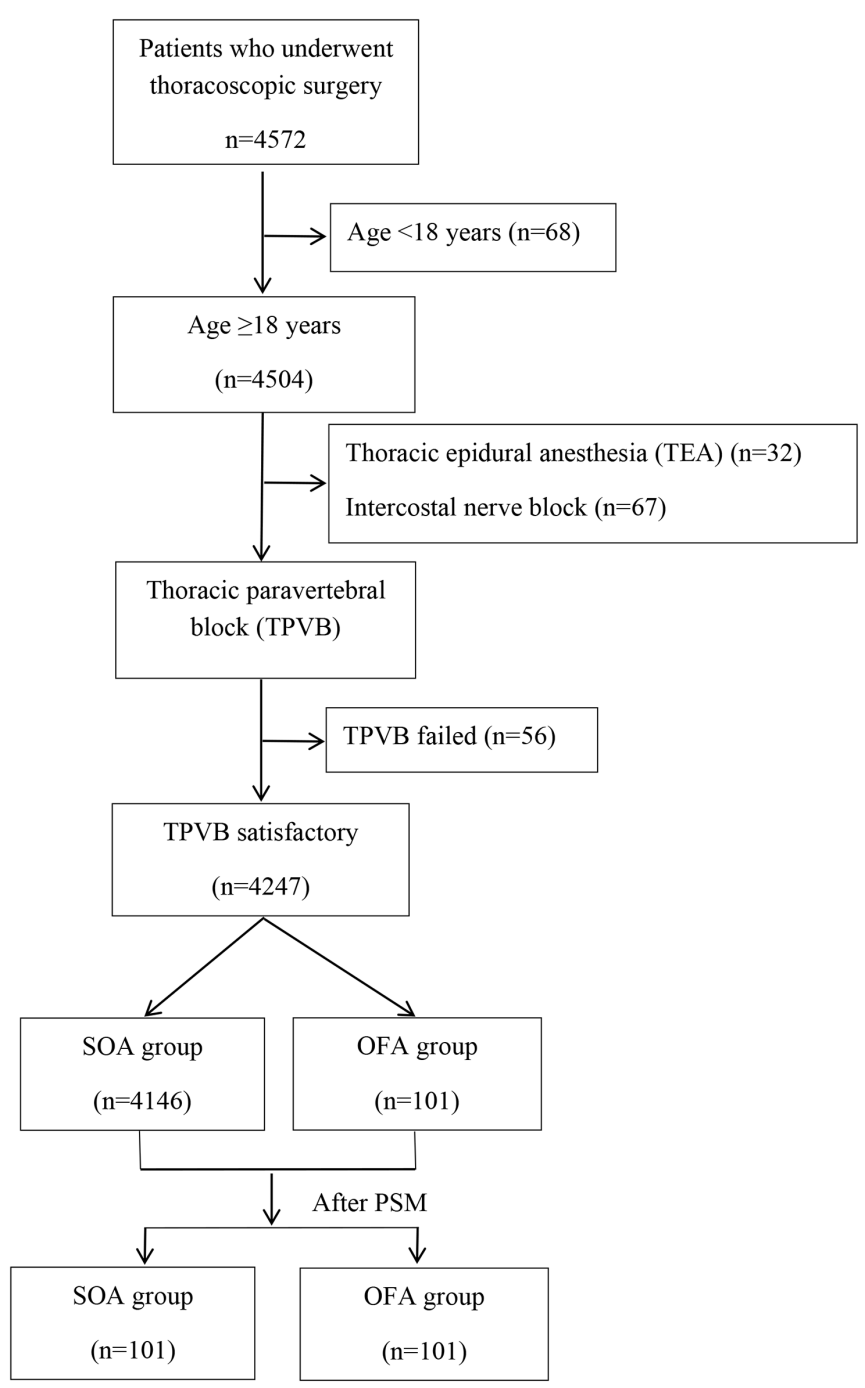
Flow chart of subjects included in the study. SOA, standard opioid-containing anaesthesia; OFA, opioid-free anaesthesia; PSM, propensity-score matching

Between January 2017 and June 2020, there were 4572 patients who underwent thoracoscopic surgery, including 68 patients aged < 18 years and 4504 patients ≥ 18 years. All 4504 adult patients received a preoperative nerve block analgesia, of which 32 received thoracic epidural anaesthesia (TEA), 67 received intercostal nerve block, 102 received erector spinae nerve block, and 4303 received TPVB. Among the 4303 patients who received TPVB, the block effect failed in 56 patients. OFA was administered to 101 patients and 4146 patients were administered SOA. After 1:1 NNM, all 101 patients in the OFA group were matched with 101 patients from the SOA group (Fig. 1). The baseline level of clinicopathological characteristics of patients before matching is shown in Table 1. After matching, there were no significant differences in clinicopathological characteristics between the two groups (Table 1).

**Table 1:** Patient characteristics before and after nearest-neighbor propensity-score matching(PSM)

	Before PSM			After PSM		
	SOA Group (*n* = 4146)	OFA Group (*n* = 101)	*P*-value	SOA Group (*n* = 101)	OFA Group (*n* = 101)	*P*-value
Age (years)	58.00 ± 15.00	57.07 ± 11.41	0.960	58.05 ± 11.27	57.07 ± 11.41	0.540
Male/female	1894/2252	52/49	0.248	51/50	52/49	0.888
BMI (kg/m^2^)	24.03 ± 4.04	24.04 ± 3.07	0.518	23.87 ± 2.80	24.04 ± 3.07	0.684
ASA (n (%))			0.122			0.489
I	786 (19.0)	13 (12.9)		17 (16.8)	13 (12.9)	
II	3147 (76.0)	86 (85.1)		80 (79.2)	86 (85.1)	
III	211 (5.0)	2 (2.0)		4 (4.0)	2 (2.0)	
IV	2 (0.0)	0 (0.0)		0 (0.0)	0 (0.0)	
HBP (yes/no)	1164/2982	28/73	0.938	27/74	28/73	0.874
Classification of surgery (n (%))			0.297			0.807
Wedge resection	1251 (30.2)	40 (39.60)		39 (38.61)	40 (39.60)	
Segmentectomy	149 (3.6)	3 (2.97)		4 (3.96)	3 (2.97)	
Lobectomy	2277 (54.9)	50 (49.51)		50 (49.51)	50 (49.51)	
Sleeve pneumonectomy	13 (0.3)	0 (0)		0 (0)	0 (0)	
Unilateral pneumonectomy	15 (0.4)	1 (0.99)		0 (0)	1 (0.99)	
Thymic mediastinal surgery	441 (10.6)	7 (6.93)		8 (7.92)	7 (6.93)	
Intraoperative bleeding volume (mL)	30 [20;50]	20 [20;50]	0.024	20 [15;30]	20 [20;50]	0.356

Data are presented as median [Interquartile range] or mean ± standard deviation or number (%) of patients

BMI, body mass index; ASA, American Society of Anesthesiologists; OFA, opioid-free anesthesia; HBP, high blood pressure; SOA, standard opioid-containing anesthesia

**Fig. 2:**
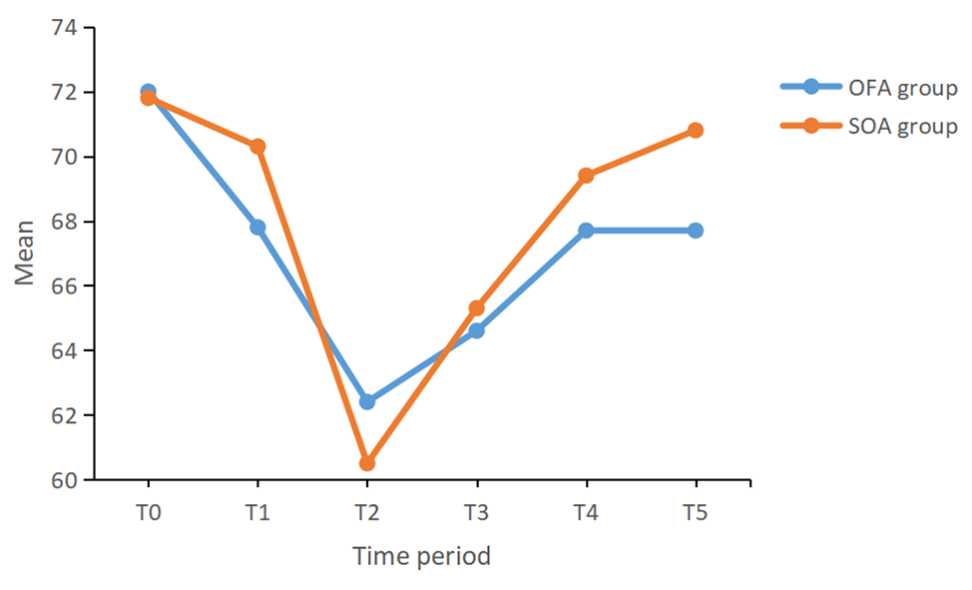
Intraoperative HR changes of patients in the two groups (F = 0.889, *P* = 0.347)

**Fig. 3:**
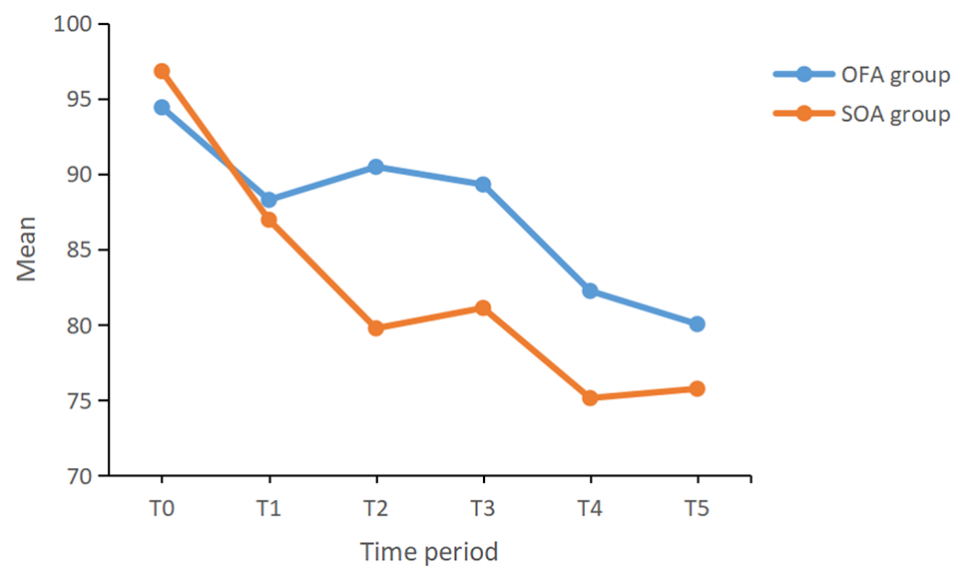
Intraoperative MAP changes of patients in the two groups (F = 16.709, *P* < 0.001). SOA, standard opioid-containing anaesthesia; OFA, opioid-free anaesthesia; T0, before anaesthesia induction; T1, immediately after endotracheal intubation; T2, at the beginning of surgery; T3, 10 min after surgery began; T4, 20 min after surgery began; T5, 30 min after surgery began

Figures 2 and 3 show the changes in HR and MAP in both groups. There was no significant difference in the changes in HR between the two groups (*P* = 0.347). However, there was a significant difference in the changes in MAP between the two groups (*P* < 0.001). Compared with SOA group, the fluctuation of MAP in the OFA group was smaller and significantly higher.

The clinical results are displayed in Table 2. There was a significant difference in the frequency of vasoactive drug use between the two groups (*P* = 0.001) and in intraoperative infusion volume between the two groups (*P* = 0.001). There was a significant difference in the morphine consumption in the PCIA pump 24 h post-surgery between the two groups ( *P* < 0.001). There was no significant difference in VAS scores at rest (*P* = 0.745) or during activity (*P* = 0.792) between groups on the first day post-surgery. For nausea and vomiting within 24 h post-surgery, there was no statistically significant difference (*P* = 0.651). In the SOA group, the opioids consumed were remifentanil (81.6 ± 28.38 µg) and sufentanil (23.2 ± 8.85 µg). However, data on single administration of remifentanil was obtained, and the total amount of remifentanil pumped continuously during the procedure was not obtained.

**Table 2:** Clinical results

	SOA Group (*n* = 101)	OFA Group (*n* = 101)	Z/χ^2^ Value	*P*-value
Frequency of vasoactive drugs			19.623	0.001
0	44 (43.6)	70 (69.3)		
1	36 (35.6)	24 (23.8)		
2	18 (17.8)	4 (3.9)		
3	3 (3.0)	2 (2.0)		
4	0 (0.0)	1 (1.0)		
Intraoperative infusion volume (mL)	1000 [1000;1500]	1000 [1000;1000]	3.353	0.001
24-h morphine PCIA use (mg)	3.6 [0.6;23]	1.8 [0;4.8]	3.537	< 0.001
VAS score on D1				
VAS (at rest)	1 [0;2]	1 [0;1]	0.325	0.745
VAS (at activity)	3 [2;4]	3 [2;3]	0.264	0.792
Frequence of nausea and vomit			0.205	0.651
0	99(98.0)	98(97.0)		
1	2(2.0)	3(3.0)		
Intraoperative opioid dosage				
Remifentanil^a^	81.6 ± 28.38 µg			
Sufentanil	23.2 ± 8.85 µg			

Data are presented as median [Interquartile range] or mean ± standard deviation or number (%) of patients.^a^The remifentanil dose did not include the intraoperative maintenance dose

PCIA, patient-controlled intravenous analgesia; VAS, visual analogue score; D1, first day after surgery

## Discussion

Opioids have been an integral part of balanced anaesthesia because of their strong analgesic effect. They can also inhibit sympathetic nerve excitation and do not cause histamine release. Therefore, they can inhibit the stress response caused by various noxious stimuli brought about by surgery during general anaesthesia [3], to maintain the stability of patient haemodynamics during surgery. However, with the recognition of adverse reactions to opioids and the promotion of the ERAS concept, OFA has been increasingly studied and reported, with its the safety and effectiveness a focus of attention. Thus it is necessary to collect relevant data to clearly evaluate the benefit–risk ratio of OFA.

Many factors affect hamodynamic changes during surgery. Therefore, we adopted NNM and included basic characteristics of the patients such as sex, age, BMI, ASA grade, presence or absence of hypertension, type of operation, and amount of intraoperative bleeding in the matching factors. After matching, there were no significant differences between the two groups in these characteristics. In a review on OFA in thoracic surgery, Tempe and Sawhney [3] reported that an important factor contributing to successful OFA is regional anaesthesia or nerve block, such as TEA, TPVB, or intercostal nerve block. TEA was once considered the gold standard for postoperative analgesia in thoracic surgery [14], but has significant side effects, including hypotension, respiratory depression and urinary retention; moreover, rare and related complications may result in permanent nerve damage. Several studies [15–18] have shown that TPVB can provide the same analgesic effect as TEA, with milder side effects such as hypotension and respiratory depression. A previous review [19] also found that for patients with normal blood volume, the occurrence of hypotension with TPVB was less common compared with TEA, which was attributed to unilateral sympathetic block. Moreover, Scarci et al. [20] found that TPVB reduced the incidence of hypotension and bradycardia compared with TEA.

In this study, the patients in both groups received preoperative TPVB, which affected intraoperative blood pressure less while reducing the stress response and provided a superior analgesic effect [14]. However, compared with the patients in the SOA group, those in the OFA group had less fluctuation in intraoperative MAP (Fig. 3). In addition, patients in the SOA group had higher rates of intraoperative hypotension and used pressor drugs more frequently. Therefore, the influence of other intraoperative factors on blood pressure should be considered, and the most important difference between the two groups was the use of opioids.

Previous studies have shown that sufentanil and remifentanil can directly expand peripheral blood vessels while inhibiting the sympathetic nerve [21] in a dose-dependent manner; this may be why the incidence of intraoperative hypotension in the SOA group was higher than that in the OFA group. In addition, there was a significant difference in intraoperative infusion volume between groups. For patients who were fasting for a long time, the anaesthesiologist accelerated the infusion speed and supplemented blood volume as treatment measures to ensure the stability of the patient’s haemodynamics, in addition to using vasoactive drugs.

Kamdar et al. [22] and Mulier and Dekock [23] suggested that OFA can effectively reduce the postoperative dosage of opioids. In terms of postoperative analgesia, the present study also conducted a correlation analysis. There was a significant difference between groups in the consumption of morphine by the PCIA pump 24 h post-surgery. Consumption in the OFA group was significantly lower than in the SOA group, indicating that OFA could improve early postoperative analgesia and reduce the dose of postoperative opioids; this may be due to the pain sensitization caused by intraoperative opioids. Joly et al. [24] also found that opioids might increase the area of secondary hyperalgesia around the surgical wound, thus increasing the demand for postoperative opioids, even if they did not improve the postoperative pain score. In addition, the acute tolerance of postoperative opioids (opioid-induced hyperalgesia) is correlated with the dosage of intraoperative opioids, which may aggravate the demand for opioid analgesics for postoperative pain [8]. Studies have shown that high-dose intraoperative remifentanil [25] or sufentanil [26] can aggravate postoperative pain. However, the dose of remifentanil and sufentanil in the study were not considered a high dose, but due to incomplete statistical data of remifentanil, the impact of intraoperative opioid dosage on the acute tolerance of postoperative opioids may require further research. Relevant clinical trials have shown that intravenous lidocaine can reduce postoperative pain, and the combination of dexmedetomidine can further enhance this effect [27, 28]. This may be another reason why the OFA group effectively reduced the dosage of postoperative analgesic drugs.

Moreover, this study found no significant difference in VAS scores at rest or during activity between groups on the first day post-surgery. This was also the case for nausea and vomiting within 24 h post-surgery. This study suggested that, although there was a significant difference in the consumption of morphine by the PCIA pump 24 h post-surgery, this was not clinically relevant.

### Limitations

This study has some limitations. First, since it was a retrospective cohort study, it was limited by a lack of blinding; therefore, there was potential for bias. In addition, a large clinical effect would be required from any intervention to demonstrate a statistically significant difference between study groups.Second, although propensity-score matching may have assisted in accounting for observed differences between the two groups, it cannot account for unobserved differences and therefore leaves room for residual confounding. Third, the difference between the two groups was not only in the use or non-use of opioids, and the anaesthesia method in the two groups was different in many ways. For example, in the SOA group, the anaesthesia methods may also be different.Therefore, the difference in the results of the study cannot be attributed solely to opioid drugs and need to confirm in a larger, more robust trial.However, the findings of this study provide a basis for further prospective randomised controlled trials.

## Conclusion

This study demonstrated that during thoracoscopic surgery, patients managed with OFA were more stable in terms of MAP than patients who were managed with SOA. Although OFA reduced morphine consumption via the PCIA pump 24 h post-surgery, postoperative pain scores and nausea and vomiting within 24 h post-surgery were similar between the groups. In conclusion,OFA in thoracoscopic surgery is safe and feasible and, at the very least, appears to be noninferior to standard anesthesia techniques involving opioid administration.To further elucidate these potential benefits, a prospective, randomised controlled trial would be necessary.

## Data Availability

The datasets used and analysed during the current study are available from. the corresponding author on reasonable request.

## Abbreviations

ERAS: Enhanced recovery after surgery
OFA: Opioid-free anaesthesia
PONV: Postoperative nausea and vomiting
TPVB: Thoracic paravertebral block
PCIA: Patient-controlled intravenous analgesia
ICU: Intensive care unit
HR: Heart rate
MAP: Mean arterial blood pressure
VAS: Visual analogue scale
BIS: Bispectral index
PACU: Postanesthesia care unit
SOA: Standard opioid-containing anaesthesia
ASA: American Society of Anesthesiologists
BMI: Body mass index
NNM: Nearest-neighbor matching
SD: Standard deviation
TEA: Thoracic epidural anaesthesia
D1: First day after surgery
HBP: High blood pressure
